# Controlling litter effects to enhance rigor and reproducibility with rodent models of neurodevelopmental disorders

**DOI:** 10.1186/s11689-020-09353-y

**Published:** 2021-01-04

**Authors:** Jessica A. Jiménez, Mark J. Zylka

**Affiliations:** 1 Curriculum in Toxicology & Environmental Medicine, The University of North Carolina at Chapel Hill, Chapel Hill, NC 27599 USA; 2 UNC Neuroscience Center, The University of North Carolina at Chapel Hill, Chapel Hill, NC 27599 USA; 3 Carolina Institute for Developmental Disabilities, The University of North Carolina at Chapel Hill, Chapel Hill, NC 27599 USA; 4 Department of Cell Biology and Physiology, The University of North Carolina at Chapel Hill, Chapel Hill, NC 27599 USA

**Keywords:** Litter effect, Rigor and reproducibility, Neurodevelopmental disorders, Animal models

## Abstract

Research with rodents is crucial for expanding our understanding of genetic and environmental risk factors for neurodevelopmental disorders (NDD). However, there is growing concern about the number of animal studies that are difficult to replicate, potentially undermining the validity of results. These concerns have prompted funding agencies and academic journals to implement more rigorous standards in an effort to increase reproducibility in research. However, these standards fail to address a major source of variability in rodent research brought on by the “litter effect,” the fact that rodents from the same litter are phenotypically more similar to one other than rodents from different litters of the same strain. We show that the litter effect accounts for 30–60% of the variability associated with commonly studied phenotypes, including brain, placenta, and body weight. Moreover, we show how failure to control for litter-to-litter variation can mask a phenotype in *Chd8*^V986*/+^ mice that model haploinsufficiency of *CHD8*, a high-confidence autism gene. Thus, if not properly controlled, the litter effect has the potential to negatively influence rigor and reproducibility of NDD research. While efforts have been made to educate scientists on the importance of controlling for litter effects in previous publications, our analysis of the recent literature (2015–2020) shows that the vast majority of NDD studies focused on genetic risks, including mutant mouse studies, and environmental risks, such as air pollution and valproic acid exposure, do not correct for litter effects or report information on the number of litters used. We outline best practices to help scientists minimize the impact of litter-to-litter variability and to enhance rigor and reproducibility in future NDD studies using rodent models.

## Introduction

Brain development requires tight coordination of cell proliferation, differentiation, migration, and synapse formation. Any disruption to this complex chain of events has the potential to perturb brain development and increase risk for a neurodevelopmental disorder (NDD), such as autism spectrum disorder, schizophrenia, intellectual disability, attention deficit hyperactivity disorder, or bipolar disorder [1–3]. Genetic and environmental factors influence risk for NDDs and do not operate alone, but rather interact to increase disease risk [4].

Research with rodent models has been crucial to expanding current knowledge of NDD risk and pathogenesis. However, a growing chorus in the scientific community has raised concerns about the number of animal studies that are difficult to replicate [5, 6], including in the preclinical NDD research field [7–9]. These concerns are important to address, as rodent models are extensively being used to further our understanding of mammalian biology and to develop treatments for human diseases. Concerns about reproducibility have touched almost every field [10]. In response, prominent institutions, including the National Institutes of Health (NIH) and the National Academy of Science (NAS), and journals, such as *Science* and *Nature*, have revised their policies to include more rigorous statistical analyses, transparency in reporting and data sharing, and greater consideration of relevant biological variables to address reproducibility concerns [5].

One variable that is well-known in the toxicology field to affect reproducibility, but that has not been consistently reported or discussed in the NDD field, is rigorous control of “litter effects” in multiparous species [11–13]. Litter effect refers to the fact that rodents from the same litter are phenotypically more similar to each other than rodents from different litters of the same strain, and this includes inbred strains which are considered to be genetically identical. While efforts have been made to shed light on the importance of litter effects [13–16], our recent literature search shows that the issue remains largely neglected in the NDD field. This review will discuss why it is important to control for litter effects and how to control for litter effects when using rodents. As we expand upon below, litter effects account for an astounding 30–60% of the variability in commonly studied phenotypes. Given that most NDD phenotypes in rodent models are of small effect size, controlling this major source of variability will go a long way towards enhancing rigor and reproducibility in the NDD field.

## Methods

### Mice

All procedures in this study were approved by the Institutional Animal Care and Use Committee at the University of North Carolina at Chapel Hill. Mice were maintained on a C57BL/6J background and raised in a facility with a 12:12 light:dark cycle with ad libitum access to food (Teklad 2020X, Envigo, Huntingdon, UK) and water. Male mice heterozygous for a high confidence *CHD8* mutation (*Chd8*^V986*/+^), generated as previously described [17], were time mated with wild-type females. Matings were set up in the evening before the start of the dark cycle, using one male mouse and one female mouse per breeding cage. Females were separated and single-housed upon confirmation of a vaginal plug the next day, considered as gestation day 0.5 (E0.5). Genotyping was performed as previously described [17]. Dams were sacrificed on E15.5, and embryos were collected by dissection in PBS. Placenta, whole body, and brain weights were determined using an analytic balance.

### Statistics and analysis

Data analysis was first based on ANOVA without adjusting for the litter effect. Litter effect was then examined by comparing the sums of squares with or without the litter as a variable in the model. To appropriately account for the litter effect, we utilized a nonlinear mixed model approach by applying the R-package “nlme” to our data, with the litter variable as a random effect.

### Literature search

Recent publications in the NDD field were identified on PubMed using the search terms shown in Table 1. Key words were selected to maximize the number of papers reviewed in particular research areas (i.e., genetic—including molecular studies of brain development, and environmental exposure studies). References were filtered for articles published between 2015 and August 26, 2020. Only primary research articles that examined mutant rodent models or the effect of prenatal environmental manipulations on rodent offspring were selected. In vitro studies were excluded. Key information extracted from each publication included whether the litter was identified as the experimental unit and whether the number of litters assessed was indicated.

**Table 1:** Litters are rarely used as the statistical unit in NDD publications

Category	Search criteria	Number of litters indicated	Litter treated as statistical unit
Genetic (including molecular studies of brain development)	(**SCN2A** AND **Autism**) OR (**CHD8** AND **Autism**) OR (**UBE3A** AND **Angelman)**	2/45 (4%)	0/45 (0%)
	**neocortex** AND **development** AND **regulation**	3/29 (10%)	1/29 (3%)
	(**embryonic knockout** OR **electroporation**) AND **brain development**	4/25 (16%)	1/25 (4%)
Genetic total		**9/99 (9%)**	**2/99 (2%)**
Environment	**maternal immune activation** AND **autism**	23/33 (67%)	6/33 (18%)
	**autism** AND **air pollution**	8/9 (89%)	3/9 (33%)
	**BPA** AND **brain development**	8/9 (89%)	4/9 (44%)
	**autism** AND **VPA**	30/66 (45%)	10/66 (15%)
Environment total		**69/117 (59%)**	**23/117 (20%)**

## Main text

Litter effects are important to control when genetic and/or environmental risks are studied in multiparous animals, particularly when the manipulation has the potential to impact animals prenatally and/or early postnatally prior to weaning. In exposure studies, it is common to apply treatments to whole litters by manipulating a pregnant dam (i.e., with a chemical, stress, or virus) and assessing individual offspring. The experimental unit, defined as the smallest physical unit that can be randomly assigned to a treatment condition, is the pregnant dam. Thus, the statistical unit of measure should be the litter. Likewise, if the litter (treated prenatally) is allowed to develop into adulthood, the statistical unit should still be the litter. Even in the absence of an exposure, littermates are more similar to one another across a variety of morphological, biochemical, and behavioral parameters, on average, than to animals from other litters [13, 16, 18]. As a result, it is also important to control for litter effects when comparing wild-type and mutant animals. Differences linked to litter variation have been reported from gestation into adulthood (as late as 2–4 years), supporting the persistence of litter effects late in life [16].

### Published literature largely fails to control for litter effects

To evaluate the extent to which litter effects are reported and/or controlled in the NDD field, a literature review was performed on recent publications. In our assessment of research articles on genetic studies of *Scn2a*, *Chd8*, and *Ube3a* rodent models, no (*n* = 0 out of 45) papers correctly accounted for litter effects by identifying the litter as the statistical experimental unit, and only 2 papers on molecular studies of brain development did so (Table 1). In total, 2% (2 out of 99) of the genetic studies, including molecular studies of brain development, corrected for litter effects. In contrast, 20% (23 out of 117) studies on environmental exposures, including air pollution, bisphenol A (BPA), valproic acid (VPA), and maternal immune activation, correctly identified the litter as the statistical experimental unit (Table 1). To account for the possibility that litter variation was considered but not corrected for in these studies, we also investigated whether studies reported on the numbers of litters used, as this is the first step when considering litter variation. Using this looser definition, 59% of environmental studies reported on litters used, only 9% of genetic studies did so (Table 1).

Almost all prenatal exposure studies investigate embryonic and early postnatal timepoints, while 35% the genetic studies assessed in this review reported on adult rodent models only. It is evident that litter effects can be more obvious in embryonic studies, due to difficulties in precisely timing conception. Thus, researchers investigating early life timepoints may take litter effects more seriously, to minimize erroneous inferences caused by sampling animals from a small number of litters. To evaluate whether genetic studies assessing embryonic timepoints report more frequently on numbers of litters used, all genetic studies reporting solely on adult time points were excluded. This filter, however, only increased the percentage of genetics studies that corrected for litter effects from 2 to 3% and increased the rate of studies reporting the numbers of litters used from 9 to 14% (data not shown).

We speculate that almost no molecular/genetic NDD studies considered litter effects, whereas a small proportion of environmental risk NDD studies did so because awareness of this issue is greater for scientists who study environmental risks. Several high-quality papers describe litter effects and the need to control litter effects when examining environmental exposures [14, 15], but would likely have been overlooked by scientists studying genetic risks. For those who are aware of litter effects, we speculate some may choose to sample a small number of litters, and hence underpower their studies, to save time, effort, and money. Awareness of the litter effect remains low overall, given that the last review of this topic by Lazic and colleagues was published in 2013 [15], yet the vast majority of recent publications failed to control for litter effects (Table 1). More work must clearly be done to educate the NDD field about this important and readily controlled source of experimental variability.

**Fig. 1:**
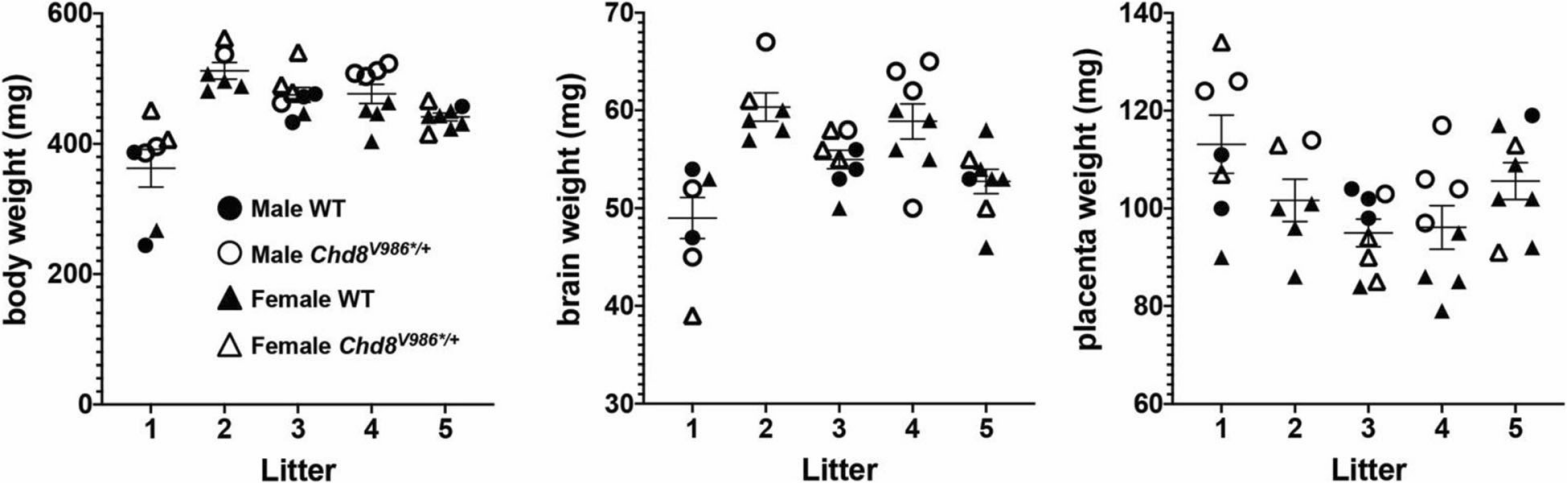
Body weight, brain weight, and placental weight of wild-type and *Chd8*^V986*/+^ mutant mice at E15.5. The variation across different litters is greater than the variation within a litter

We also speculate that NDD researchers who study rodent models with gene mutations may believe that litter effects only need to be considered if studying embryonic environmental exposures. However, this is not the case. Studies with animals harboring a mutant allele from conception onward are essentially no different than studies that expose animals to a candidate environmental risk. In both cases, an experimental manipulation is being evaluated that has the potential to influence brain development in the pre- and/or early postnatal period. As a case in point, our data with wild-type and *Chd8*^V986*/+^ mice, which model a high-confidence autism gene mutation [17], indicate that within litter variation is lower than between litters (Fig. 1).

### Appropriate analysis to remove the influence of litter effects

**Fig. 2:**
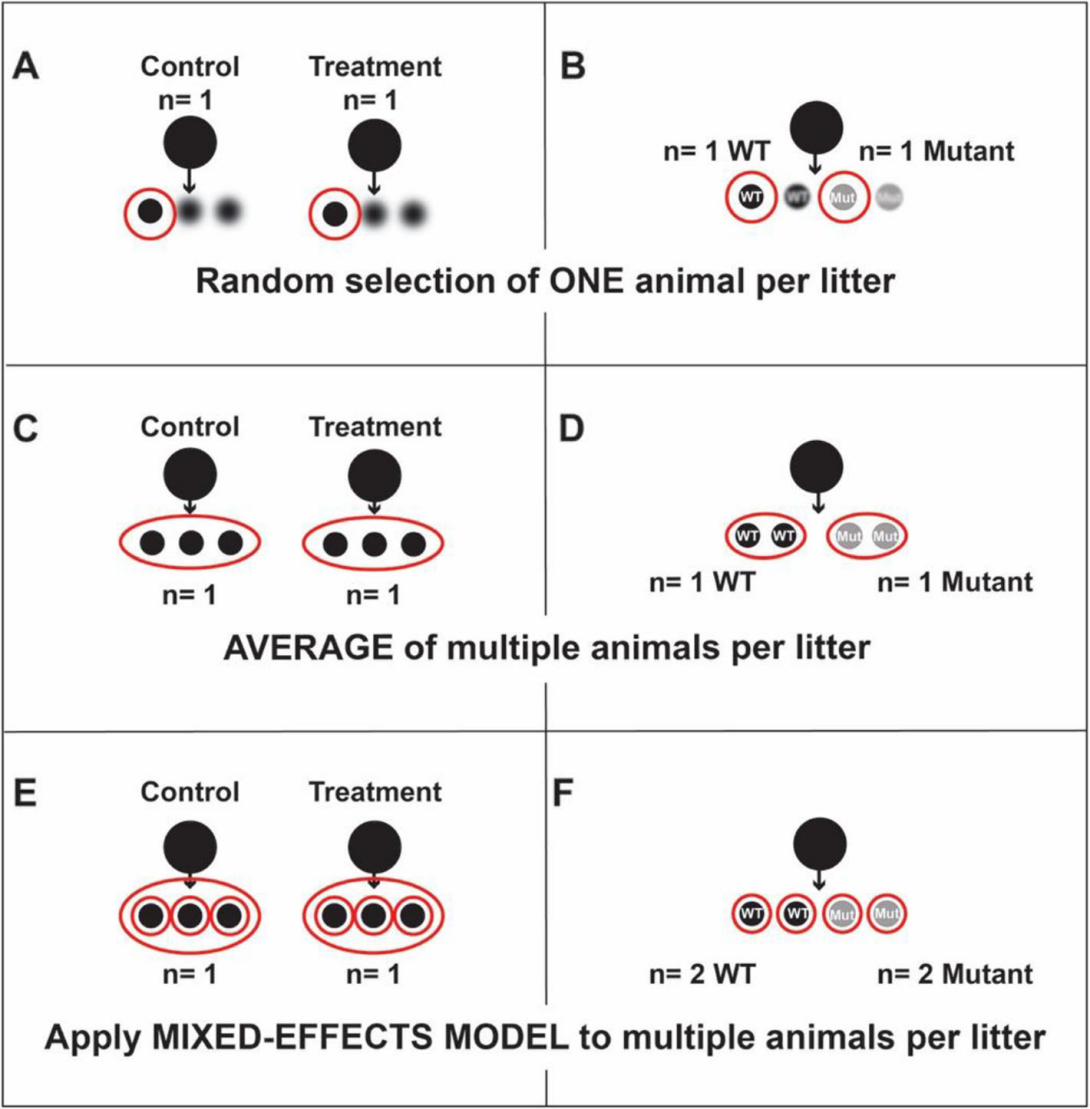
In treatment studies, pregnant females, and hence whole litters, are exposed to a substance (**a**, **c**, **e**). Litters are the experimental unit because they are randomized to the treatment. Offspring within a litter will be more similar to each other than to offspring from different litters and should be treated as subsamples or technical replicates. Similarly, in studies with mutant animals, individual animals coming from the same litter share prenatal and postnatal environments that render them more similar to each other than to animals from different litters (**b**, **d**, **f**). Most studies incorrectly ignore the litter and instead apply standard statistical tests to multiple animals per litter, thus failing to correct or control for relatively large differences between litters. Appropriate analysis can be conducted by **a** and **b** only using one animal of a given genotype and sex per litter (randomly selected), **c** and **d** using more than one animal per litter and averaging their values, or **e** and **f** using multiple animals per litter and applying a mixed-effects model for analysis. The mixed-effects model allows for each offspring to be used as a unit of analysis and treats the litter as a random variable in the ANOVA. Even though each offspring is used as a sample, the litter is still the statistical unit for exposure studies, as the dam was randomly assigned to the treatment condition. Sex of the offspring should be balanced across groups. It may be necessary to study male and female neonates separately, in which case a total of 1 male and 1 female per litter (and genotype if relevant), can be sampled

Research with rodent models must account for litter effects in the experimental design and statistical analysis. Appropriate design and analysis can be conducted by (1) only using one animal per litter (randomly selected), (2) using more than one animal per litter and averaging their values, or (3) using multiple animals per litter and applying a mixed-effects model for analysis (Fig. 2) [15]. This contrasts with the evidently common practice of using multiple wild-type and/or mutant mice or rats from a small number of litters (Table 1), which erroneously inflates sample size and fails to correct for large litter-to-litter variation.

Lazic and Essioux nicely describe the benefits of each method [15]. Briefly, while using one animal per litter allows standard statistical methods, such as *t* tests and ANOVA, to be used for analysis, it is not an efficient use of animals unless one plans additional experiments, analyzing different endpoints, with the other animals in the litter. Standard statistical methods can also be performed by using the average value of multiple animals per litter. However, the precision of estimated variability within a litter will be lost by averaging. Alternatively, one can assess multiple pups per litter and apply the mixed-effects model for analysis, which allows one to quantify the magnitude of the litter effect, or the variability within each litter, and remove unexplained variation in the data (Fig. 2).

**Fig. 3:**
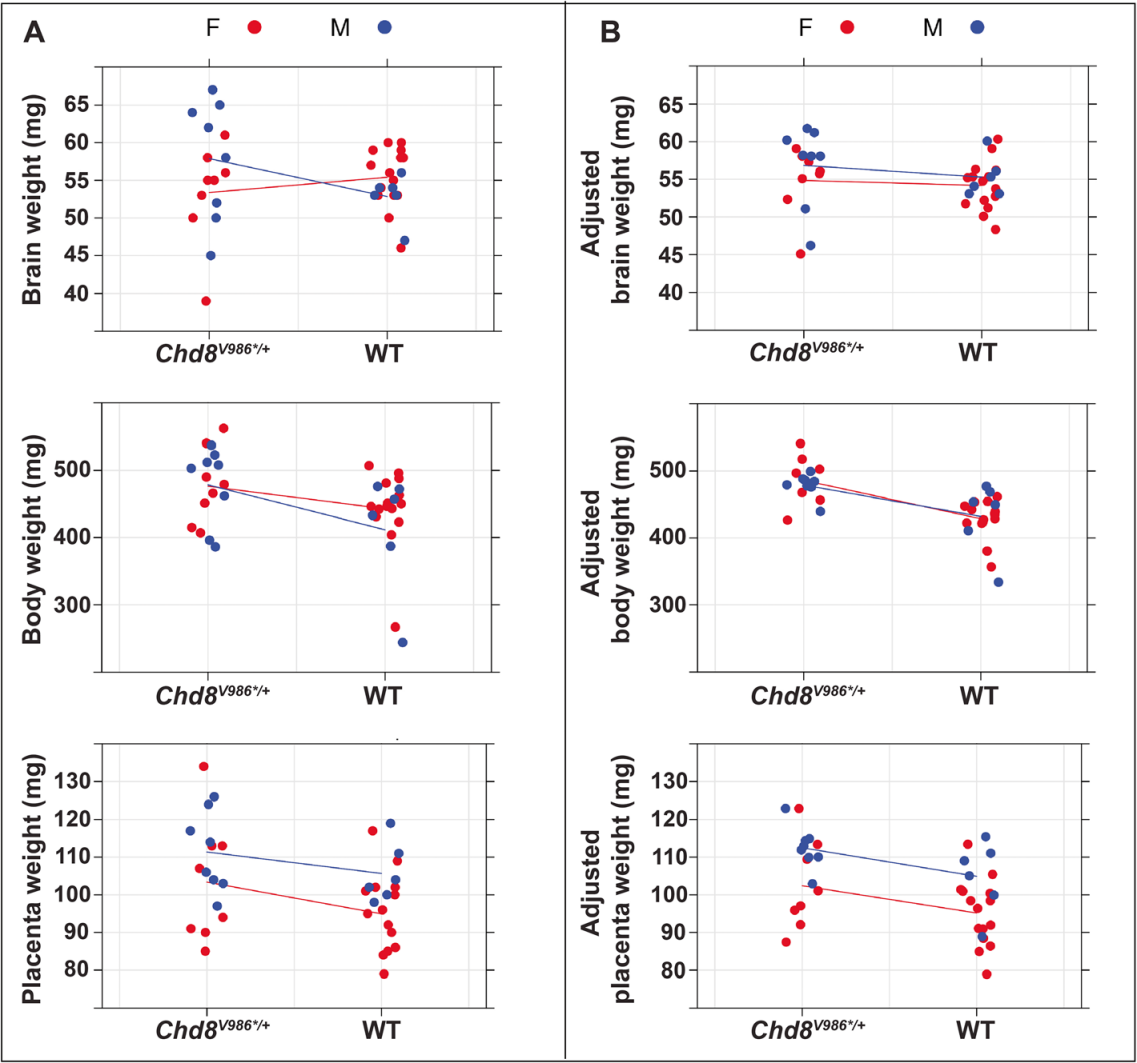
**a** When litter effect is ignored, the variation in body, brain, and placental weights of wild-type (WT) and *Chd8*^V986*/+^ mutant mice at E15.5 varies greatly. **b** Adjusting for the litter effect removes unexplained variation in the data and has the potential to unmask significant differences between groups

Experimental designs that do not appropriately account for litter effects can lead to erroneous inferences. Holson and Pearce showed that false positive rates increase as a greater number of offspring are sampled per litter [13]. Conversely, litter-to-litter variation adds “noise” to the data that can mask true treatment or genetic effects. For example, we found that body, brain, and placenta weight data were similar within litters but differed between litters dissected on embryonic day 15.5 (E15.5) from wild-type and *Chd8*^V986*/+^ mutant mice (Fig. 1). When litters were included as a variable, and hence litter effect was controlled, the effects of sex and genotype increased in significance, becoming more evident than was observed by treating each pup as an individual statistical unit (Table 2, Fig. 3). For example, male placenta weight was significantly greater than female placenta weight (Table 2, Fig. 3), consistent with prior studies [19–21]. Importantly, the statistical significance of this measure increased after correcting for litter-to-litter variation. Further, litter effects were found to account for 53.4%, 63.7%, and 34.4% of variation in brain weight, body weight, and placental weight, respectively (Table 2, Fig. 3).

**Table 2:** Litter effect reflected in E15.5 body and organ weights of wild-type and *Chd8*^V986*/+^ mutant mice

	Standard analysis (litter effect ignored)	Mixed-effects model
**Brain:**		
Sex	0.606	0.476
Genotype	0.695	0.587
Variation in brain weights explain by litter effect: **53.4%**		
**Body:**		
Sex	0.829	0.696
Genotype	0.032^*^	0.0003^***^
Variation in body weights explained by litter effect: **63.7%**		
**Placenta:**		
Sex	0.008^**^	0.001^**^
Genotype	0.06^#^	0.023^*^
Variation in placenta weights explained by litter effect: **34.4%**		

***p < 0.001, **p < 0.01, *p < 0.05, ^#^*p* < 0.1

While sampling multiple pups per litter allows one to quantify the litter effect and reduce its influence on the data, it is not always feasible to test every animal. In this case, increasing the litter number and only testing one animal, randomly selected, per litter will allow one to avoid falsely inflating sample size. Regardless of the model used, increasing the number of litters will increase statistical power far more than increasing the number of animals per litter. This important point was nicely depicted by Lazic and colleagues in graphical format [15], using locomotor activity to perform power calculations. Moreover, using one animal per litter and multiple litters also saves researchers time and resources, as fewer animals need to be tested overall to increase statistical power.

### Factors that contribute to litter effects

There are several major factors that likely contribute to similarities within litters and variation across litters. Animals within a litter are genetically similar, they share the same prenatal and postnatal environments, and they were all conceived at the same time—a time that cannot possibly be identical to animals in other litters, even if timed matings were used to generate the animals. Timed mating occurs overnight, typically over a 12-h window, which is hardly a short period of time given the dramatic changes that take place each day of embryonic brain development [22, 23]. These shared features within a litter are precisely what contribute to differences between litters. Other plausible contributing factors to litter effects that are seldom considered include differences in maternal behaviors, particularly if the mother harbors a mutant allele that affects maternal behaviors [17, 24], and variation within the in utero environment. These factors cannot be accounted for statistically if the litter is not controlled as a variable.

### Influence of maternal behavior on litter effects

Naturally occurring variation in maternal behavior has been reported between and within rodent strains [25–27]. C57BL/6J mice display considerable natural variation in the frequency of postpartum maternal behavior observed daily from day 1 through day 6 postpartum. Nursing frequency ranged from 37–73%, 3–14% for licking/grooming, and 1–21% for nest building [28]. Adult offspring of C57BL/6J mothers that exhibit low maternal licking display increased anxiety-like behavior, impaired habituation to testing scenarios, increased reactivity to acute stressors, and deficits in prepulse inhibition in female offspring [29].

By influencing the development of neural systems, including the hypothalamic-pituitary-adrenal axis, hypothalamic-pituitary-gonadal axis, and mesolimbic dopamine system, mother-pup interactions shape behavioral and endocrine response systems [26, 27, 30]. High nurturing behavior can also lead to increased expression of neural cell adhesion molecules and brain-derived neurotrophic factors, suggestive of increased synapse formation and neuron survival [26, 31, 32]. Thus, the behavior of the mother toward her offspring can lead to sustained changes in neuronal gene expression that influence neuroendocrine responses to stress and behavior in adulthood. Offspring of mice with high nurturing behavior also show enhanced spatial learning, memory, and object recognition [26, 32].

Differences in social environments across litters can also contribute to litter-to-litter variation. During the first weeks of life, the social environment, defined by the mother and littermates, is extremely restricted. Therefore, the mother serves as the primary and direct link between the environment and the developing pups. Early life events that prolong the activation of maternal stress reactivity, such as isolation or frequent handling, can promote vulnerability to chronic illness later in life in the offspring [26, 27]. Variation in litter size also has the potential to influence the developing offspring. Small litter size has been associated with increased body weight and impacts memory and anxiety-related behaviors in a strain-dependent manner in male mice [33]. These effects may result from greater access to nutrients or differences in maternal behavior when litters are small. Culling litters to the same number of pups, to ensure equal access to milk and to better control for pup-pup stimulation, may reduce variability. However, this practice has become controversial as increasing evidence supports long-term unforeseen consequences, including an altered feeding status that can affect metabolic functions [34, 35]. Alternatively, researchers can exclude litters outside a narrow range of litter sizes.

When using rodents to assess genetic contributors to NDDs, it is crucial to test mutant dams for early signs of maternal behavior deficits that have the potential to confound results [17]. Litter size in addition to maternal behaviors, including licking/grooming, small or absent milk spots in pups, and failure to retrieve pups should be considered [24, 36]. Deficits in these behaviors will require the fostering of pups to wild-type dams of the same strain or choosing to mate wild-type females with mutant males, if heterozygous offspring model the disorder, such is the case for most syndromic forms of autism.

### Impact of in utero environment on litter effects

Intrauterine differences in maternal hormones, nutrient availability, and environmental chemicals passing through the placenta can also affect brain development and NDD risk [13, 37–40]. For example, enhanced levels of aggression and stress during pregnancy heighten stress responses in offspring as they prepare for life outside of the womb [41–44]. Similarly, maternal malnutrition reprograms metabolism in the fetus to prepare for a life of scarcity [45, 46]. Impaired maternal cardiovascular function and maternal anemia can deprive the fetus of an adequate blood supply and result in intrauterine hypoxia. Chronic hypoxia during brain development can affect neuronal migration and profoundly affect brain development [39].

Perturbed brain development has also been associated with infection and maternal immune activation during pregnancy. Specifically, human epidemiological studies and rodent models have linked bacterial and viral infections during pregnancy to neurodevelopmental disorders, including autism and schizophrenia [47, 48]. Exogenous chemical exposures during pregnancy can also impact the developing embryo through molecular changes in the female, or can cross the placenta to directly affect fetal development. Substances such as alcohol and valproic acid have been associated with behavior impairments, transcriptional alterations, congenital malformation, and neuropsychiatric and neurodevelopmental disorders [39, 49, 50].

Consequently, while the fetal brain’s plasticity promotes survival, it also heightens vulnerability to exogenous manipulations. These risk factors can reflect differences in food, animal handling, and cage environment, and may go undetected in rodent research. The resulting impact on in utero environments can strongly contribute to litter effects. It is not feasible to control for variability in the response of a female to all of the possible biochemical changes that occur during pregnancy or to variability in the impact of an exogenous exposure. Thus, measures must be taken to account for these differences across litters.

## Conclusions

Many variables contribute to litter effects that can negatively impact reproducibility in preclinical NDD studies. In this review, we focused on design and analysis of preclinical NDD studies that use rodent models, and how this affects the validity and reproducibility of results. We assessed experimental designs in which natural litter-to-litter variation can influence the value of a measured experimental outcome and where an experimental treatment is applied to whole litters by dosing pregnant females and therefore all the offspring. Litter effects are an inherent characteristic of neurodevelopmental research with rodent models, yet are rarely controlled, creating the potential for failure to replicate. In our analysis of recent literature involving rodent models of NDDs, including genetic and molecular studies of brain development and environmental exposure studies, 88% of studies fail to indicate how litter effects were controlled, let alone acknowledge that litter effects were considered.

Litter effects are straightforward to control, and once controlled, will increase rigor and reproducibility in preclinical NDD studies. We recommend that NDD researchers adhere to the experimental designs and analyses discussed in this review, as well as other well-written reviews of this topic [13–16]. Observing these best practices will enhance the value of animal models and strengthen the conclusions obtained from NDD studies.

## Data Availability

Data generated and analyzed for the purpose of this review are available from the corresponding author upon request.

## Abbreviations

NDD: Neurodevelopmental disorder
NIH: National Institutes of Health
NAS: National Academy of Science
BPA: Bisphenol A
VPA: Valproic acid

## References

[1] Tunç B, Yankowitz LD, Parker D, Alappatt JA, Pandey J, Schultz RT (2019). Deviation from normative brain development is associated with symptom severity in autism spectrum disorder. Mol Autism.

[2] du Bois TM, Huang XF (2007). Early brain development disruption from NMDA receptor hypofunction: relevance to schizophrenia. Brain Res Rev.

[3] Dark C, Homman-Ludiye J, Bryson-Richardson RJ (2018). The role of ADHD associated genes in neurodevelopment. Dev Biol.

[4] Bale TL, Baram TZ, Brown AS, Goldstein JM, Insel TR, McCarthy MM (2010). Early life programming and neurodevelopmental disorders. Biol Psychiatry.

[5] Kafkafi N, Agassi J, Chesler EJ, Crabbe JC, Crusio WE, Eilam D (2018). Reproducibility and replicability of rodent phenotyping in preclinical studies. Neurosci Biobehav Rev.

[6] Begley CG, Ioannidis JP (2015). Reproducibility in science: improving the standard for basic and preclinical research. Circ Res.

[7] Sonzogni M, Wallaard I, Santos SS, Kingma J, du Mee D, van Woerden GM (2018). A behavioral test battery for mouse models of Angelman syndrome: a powerful tool for testing drugs and novel Ube3a mutants. Mol Autism.

[8] Jiang YH, Ehlers MD (2013). Modeling autism by SHANK gene mutations in mice. Neuron..

[9] Han Q, Kim YH, Wang X, Liu D, Zhang ZJ, Bey AL (2016). SHANK3 deficiency impairs heat hyperalgesia and TRPV1 signaling in primary sensory neurons. Neuron..

[10] National Academies of Sciences E, and Medicine, Policy and Global Affairs; Committee on Science, Engineering, Medicine, and Public Policy, Board on Research Data and Information, Division on Engineering and Physical Sciences, Committee on Applied and Theoretical Statistics, Board on Mathematical Sciences and Analytics, Division on Earth and Life Studies, Nuclear and Radiation Studies Board, Division of Behavioral and Social Sciences and Education, Committee on National Statistics, Board on Behavioral, Cognitive, and Sensory Sciences, Committee on Reproducibility and Replicability in Science (2019). Reproducibility and replicability in science.

[11] Kupper LL (2006). Litter Effect. Encyclopedia of Environmetrics.

[12] Weil CS (1970). Selection of the valid number of sampling units and a consideration of their combination in toxicological studies involving reproduction, teratogenesis or carcinogenesis. Food Cosmet Toxicol.

[13] Holson RR, Pearce B (1992). Principles and pitfalls in the analysis of prenatal treatment effects in multiparous species. Neurotoxicol Teratol.

[14] Festing MF (2006). Design and statistical methods in studies using animal models of development. ILAR J.

[15] Lazic SE, Essioux L (2013). Improving basic and translational science by accounting for litter-to-litter variation in animal models. BMC Neurosci.

[16] Zorilla EP (1997). Multiparous species present problems (and possibilities) to developmentalists. Dev Psychobiol.

[17] Jiménez JA, Ptacek TS, Tuttle AH, Schmid RS, Moy SS, Simon JM (2020). Chd8 haploinsufficiency impairs early brain development and protein homeostasis later in life. Mol Autism.

[18] Chapman RH, Stern JM (1979). Failure of severe maternal stress or ACTH during pregnancy to affect emotionality of male rat offspring: implications of litter effects for prenatal studies. Dev Psychobiol.

[19] Braun AE, Carpentier PA, Babineau BA, Narayan AR, Kielhold ML, Moon HM (2019). “Females Are Not Just ‘Protected’ Males”: sex-specific vulnerabilities in placenta and brain after prenatal immune disruption. eNeuro.

[20] Ishikawa H, Seki R, Yokonishi S, Yamauchi T, Yokoyama K (2006). Relationship between fetal weight, placental growth and litter size in mice from mid- to late-gestation. Reprod Toxicol.

[21] Kalisch-Smith JI, Simmons DG, Pantaleon M, Moritz KM (2017). Sex differences in rat placental development: from pre-implantation to late gestation. Biol Sex Differ.

[22] Rubenstein JL (2011). Annual Research Review: Development of the cerebral cortex: implications for neurodevelopmental disorders. J Child Psychol Psychiatry.

[23] Polleux F, Dehay C, Kennedy H (1997). The timetable of laminar neurogenesis contributes to the specification of cortical areas in mouse isocortex. J Comp Neurol.

[24] Bartsch VB, Lord JS, Diering GH, Zylka MJ (2020). Mania- and anxiety-like behavior and impaired maternal care in female diacylglycerol kinase eta and iota double knockout mice. Genes Brain Behav.

[25] Stern JM (1997). Offspring-induced nurturance: animal-human parallels. Dev Psychobiol.

[26] Meaney MJ (2001). Maternal care, gene expression, and the transmission of individual differences in stress reactivity across generations. Annu Rev Neurosci.

[27] van Bodegom M, Homberg JR, Henckens M (2017). Modulation of the hypothalamic-pituitary-adrenal axis by early life stress exposure. Front Cell Neurosci.

[28] Champagne FA, Curley JP, Keverne EB, Bateson PP (2007). Natural variations in postpartum maternal care in inbred and outbred mice. Physiol Behav.

[29] Pedersen CA, Vadlamudi S, Boccia ML, Moy SS (2011). Variations in maternal behavior in C57BL/6 J mice: behavioral comparisons between adult offspring of high and low pup-licking mothers. Front Psychiatry.

[30] Curley JP, Champagne FA (2016). Influence of maternal care on the developing brain: mechanisms, temporal dynamics and sensitive periods. Front Neuroendocrinol.

[31] Caldji C, Tannenbaum B, Sharma S, Francis D, Plotsky PM, Meaney MJ (1998). Maternal care during infancy regulates the development of neural systems mediating the expression of fearfulness in the rat. Proc Natl Acad Sci U S A.

[32] Liu D, Diorio J, Day JC, Francis DD, Meaney MJ (2000). Maternal care, hippocampal synaptogenesis and cognitive development in rats. Nat Neurosci.

[33] Salari AA, Samadi H, Homberg JR, Kosari-Nasab M (2018). Small litter size impairs spatial memory and increases anxiety-like behavior in a strain-dependent manner in male mice. Sci Rep.

[34] Chahoud I, Paumgartten FJ (2009). Influence of litter size on the postnatal growth of rat pups: is there a rationale for litter-size standardization in toxicity studies?. Environ Res.

[35] Suvorov A, Vandenberg LN (2016). To cull or not to cull? Considerations for studies of endocrine-disrupting chemicals. Endocrinology..

[36] Chourbaji S, Hoyer C, Richter SH, Brandwein C, Pfeiffer N, Vogt MA (2011). Differences in mouse maternal care behavior - is there a genetic impact of the glucocorticoid receptor?. PLoS One.

[37] Kinsella MT, Monk C (2009). Impact of maternal stress, depression and anxiety on fetal neurobehavioral development. Clin Obstet Gynecol.

[38] Weinstock M (2005). The potential influence of maternal stress hormones on development and mental health of the offspring. Brain Behav Immun.

[39] Hwang HM, Ku RY, Hashimoto-Torii K (2019). Prenatal environment that affects neuronal migration. Front Cell Dev Biol.

[40] vom Saal F, Bronson F (1980). Sexual characteristics of adult female mice are correlated with their blood testosterone levels during prenatal development. Science..

[41] Glover V (2011). Annual Research Review: Prenatal stress and the origins of psychopathology: an evolutionary perspective. J Child Psychol Psychiatry.

[42] Van den Bergh BR, Mulder EJ, Mennes M, Glover V (2005). Antenatal maternal anxiety and stress and the neurobehavioural development of the fetus and child: links and possible mechanisms. A review. Neurosci Biobehav Rev.

[43] Talge NM, Neal C, Glover V (2007). Antenatal maternal stress and long-term effects on child neurodevelopment: how and why?. J Child Psychol Psychiatry.

[44] Beydoun H, Saftlas AF (2008). Physical and mental health outcomes of prenatal maternal stress in human and animal studies: a review of recent evidence. Paediatr Perinat Epidemiol.

[45] Brown AS, van Os J, Driessens C, Hoek HW, Susser ES (2000). Further evidence of relation between prenatal famine and major affective disorder. Am J Psychiatry.

[46] Dana K, Finik J, Koenig S, Motter J, Zhang W, Linaris M (2019). Prenatal exposure to famine and risk for development of psychopathology in adulthood: a meta-analysis. J Psychiatry Psychiatr Disord.

[47] Patterson PH (2011). Maternal infection and immune involvement in autism. Trends Mol Med.

[48] Choi GB, Yim YS, Wong H, Kim S, Kim H, Kim SV (2016). The maternal interleukin-17a pathway in mice promotes autism-like phenotypes in offspring. Science..

[49] Bilbo SD, Block CL, Bolton JL, Hanamsagar R, Tran PK (2018). Beyond infection - maternal immune activation by environmental factors, microglial development, and relevance for autism spectrum disorders. Exp Neurol.

[50] Hollander JA, Cory-Slechta DA, Jacka FN, Szabo ST, Guilarte TR, Bilbo SD (2020). Beyond the looking glass: recent advances in understanding the impact of environmental exposures on neuropsychiatric disease. Neuropsychopharmacology..

